# A Vis/NIR spectra-based approach for identifying bananas infected with *Colletotrichum musae*

**DOI:** 10.3389/fpls.2023.1180203

**Published:** 2023-06-02

**Authors:** Xuan Chu, Kun Zhang, Hongyu Wei, Zhiyu Ma, Han Fu, Pu Miao, Hongzhe Jiang, Hongli Liu

**Affiliations:** ^1^ College of Mechanical and Electrical Engineering, Zhongkai University of Agriculture and Engineering, Guangzhou, China; ^2^ College of Engineering, South China Agricultural University, Guangzhou, China; ^3^ College of Mechanical and Electronic Engineering, Nanjing Forestry University, Nanjing, China

**Keywords:** Vis/NIR spectra, banana fruit, *Colletotrichum musae* infection, fungi contamination detection, traditional classification methods, deep learning algorithms

## Abstract

**Introduction:**

*Anthracnose* of banana caused by *Colletotrichum species* is one of the most serious post-harvest diseases, which can cause significant yield losses. Clarifying the infection mechanism of the fungi using non-destructive methods is crucial for timely discriminating infected bananas and taking preventive and control measures.

**Methods:**

This study presented an approach for tracking growth and identifying different infection stages of the *C. musae* in bananas using Vis/NIR spectroscopy. A total of 330 banana reflectance spectra were collected over ten consecutive days after inoculation, with a sampling rate of 24 h. The four-class and five-class discriminant patterns were designed to examine the capability of NIR spectra in discriminating bananas infected at different levels (control, acceptable, moldy, and highly moldy), and different time at early stage (control and days 1-4). Three traditional feature extraction methods, i.e. PC loading coefficient (PCA), competitive adaptive reweighted sampling (CARS) and successive projections algorithm (SPA), combining with two machine learning methods, i.e. partial least squares discriminant analysis (PLSDA) and support vector machine (SVM), were employed to build discriminant models. One-dimensional convolutional neural network (1D-CNN) without manually extracted feature parameters was also introduced for comparison.

**Results:**

The PCA-SVM and·SPA-SVM models had good performance with identification accuracies of 93.98% and 91.57%, 94.47% and 89.47% in validation sets for the four- and five-class patterns, respectively. While the 1D-CNN models performed the best, achieving an accuracy of 95.18% and 97.37% for identifying infected bananas at different levels and time, respectively.

**Discussion:**

These results indicate the feasibility of identifying banana fruit infected with *C. musae* using Vis/NIR spectra, and the resolution can be accurate to one day.

## 1 Introduction

As a nutritious kind of fruit, bananas are vulnerable to pathogenic micro-organism and saprophyte attacks during storage and transport in the post-harvest period (Li et al., 2019; Cho and Koseki, 2021). Banana anthracnose caused by *Colletotrichum musae (C. musae*) is one of the most aggressive post-harvest fungal diseases. Fruit usually become infected in the field, and the infection develops after harvest (Khaliq et al., 2019). Anthracnose symptoms involve black or sunken brown lesions on the peel, and even finger or crown rot (Thangavelu et al., 2004). Even if only a few fruit are infected, the disease can spread rapidly to other bananas, leading to serious quality losses (Lorente et al., 2015). Previous publications have indicated that the disease can cause 30–40% losses of the marketable fruit, and even up to 80% in some cases (Vilaplana et al., 2018; Maqbool et al., 2021). Therefore, timely, rapid, and accurate recognition of this disease is crucial for guaranteeing fruit quality.

However, identifying the disease in bananas at an early stage is still challenging. Several laboratory methods, such as microbiological and/or physicochemical techniques, are accurate but they are usually slow, labor-intensive, costly and need complicated sample pretreatment (Yao et al., 2008; Alander et al., 2013; Sun et al., 2023). These compelled non-destructive and economic methods for the early detection of fungal infection (Brosnan and Sun, 2004). In recent years, a series of non-destructive methods have been employed, including soft X-ray imaging (Pearson and Wicklow, 2006), dielectric (Li et al., 2013), electronic nose (Liu et al., 2018) and hyperspectral imaging technology (Yeh et al., 2016). Although these methods showed good performance, there are still some limitations in commercial application, such as ionizing radiation, difficulty in data processing and high costs (Haff and Toyofuku, 2008; Gu et al., 2017). Visible and near-infrared (Vis/NIR) spectroscopy is an alternative method, which are faster (approximately a full spectral reading per second), easier-to-use, and lower-cost advantages (Magwaza et al., 2012; De Azevedo et al., 2019). This technique has been widely used for evaluating fruit quality, such as taste parameters (Li et al., 2019a), internal quality attributes (Chandrasekaran et al., 2019), maturity (Shah et al., 2020), and mechanical or insect damage (Moscetti et al., 2014; Nturambirwe et al., 2023). It can record the multi-frequency and co-frequency information of organic molecules (e.g., C–H, N–H, C–O, and O–H) that are related to the internal components. Sample tissue’s physical state (e.g., density, cell structures, or cellular matrices) (Cen et al., 2013; Costabile et al., 2013) also can be represented from the spectral scatter. During fungi infecting, both internal compositions and external attributes will be changed. The hypha pierces the cuticle and cell wall, then secretes large amounts of cell wall-degrading enzymes to destroy the cell structure, thus causing the fruit tissue to become soft (Sun et al., 2020). Meanwhile, fungi exploit fruit as living substrates and consume nutrients, such as chlorophyll, moisture, and saccharides (Mendgen and Hahn, 2002). The consequent changes in microstructure of tissue and chemical composition can affect the infrared radiation. Therefore, NIR spectra has a great potential in detection of the fungi infection.

Many cases of fungal contamination in fruit using NIR spectra have been reported (Huang et al., 2017; Najjar and Abu-Khalaf, 2021), such as the detection of *Alternaria alternata* and *Penicillium digitatum* infection in orange fruit (Lorente et al., 2015; Ghooshkhaneh et al., 2023), bitter pit disease in ‘Fuji’ apples (Mogollón et al., 2021), Monilia contamination in plum (Vitalis et al., 2021), moldy core in apple and pears (Zhang et al., 2022; Zhang et al., 2022a). In addition, several researchers have been aimed to identify fruit or plant anthracnose caused by *Colletotrichum* spp. infection using NIR spectra. For example, Lu et al. (2017) identified anthracnose crown rot in strawberry leaves. The identification was based on models established by 33 spectral vegetation indices that were selected from the VIS and NIR regions (400–2000 nm). Jiang et al. (2021) also achieved the early identification of anthracnose on strawberry leaves through the spectral fingerprint features in the 400–1000 nm range in reflectance mode, and obtained perfect accuracy (100%) and robust performance. Ardila et al. (2020) built models based on 29 significant spectral bands from spectral range of 350–1900 nm for the early detection of anthracnose in Mango, and obtained accuracies of 91–100% when characterizing healthy, asymptomatic, and diseased samples. Wu et al. (2012) explored the spectral characteristics of oil camellia canopies suffering from different severity of anthracnose using the NIR reflectance spectra of 200-1000 nm, and built a prediction model for its chlorophyll content. The above studies indicated excellent performance in detecting fruit and plant anthracnose using NIR spectra. For bananas, NIR spectra are commonly reported for detecting chemical composition attributes, such as moisture content, chlorophyll content, solid soluble content (SSC), and pH during microwave vacuum drying, ripening, or storing processes (Ali et al., 2018; Pu et al., 2018; Sripaurya et al., 2021; Ferreira et al., 2022). In contrast, few studies have focused on non-invasively screening fungi infecting banana fruit.

In the analysis of NIR spectral data, it should be noted that the spectral data usually contain hundreds or thousands of wavelength variables, implying a large amount of hidden information. Thus, particular attention should be paid to data mining and feature optimization (Cen et al., 2007; Li et al., 2013a). In recent years, deep learning algorithms, such as deep convolutional neural networks (DCNNs), have been widely used in fruit classification, as they can automatically learn and extract high-level data features (Liu et al., 2021). For instance, Rong et al. (2020) constructed a 1D-CNN model using Vis-NIR spectra to identify five cultivars of peaches, obtaining an accuracy of 94.4% on the test data set. Tian et al. (2022) analyzed freezing damage in orange using transmission NIRs with a 1D-CNN model. Similarly, Chen et al. (2019) proposed an end-to-end 1D-CNN model based on near-infrared spectral data to detect aristolochic acids and their analogues in Chinese herbs. In other reports, 1D-CNN models based on spectra have also used for detecting maize kernels contaminated with fungi (Mansuri et al., 2022), prediction of specialty coffee flavors (Chang et al., 2021), and geographical origin identification of Chinese chestnuts (Li et al., 2021). Therefore, in the analysis of banana fruit infected with fungi, the use of such a deep learning-based model would be a good attempt.

The aim of this study was to utilize Vis/NIR spectra to track the infection of the *C. musae* in bananas and determine the identifiable time node. The specific objectives were to: (1) classify fungal infection levels on bananas qualitatively; (2) explore the feasibility of discriminant fungal infection time and determine the earliest identifiable time node; (3) extract the informative spectral features based on traditional machine learning methods and deep learning methods.

## 2 Materials and methods

### 2.1 Sample preparation

Considering the uncontrollability and unpredictability of the fungi contamination in natural environment, it is rather difficult to directly apply the NIR spectra on detection of samples contaminated naturally. This study started with the identification of different stages of banana fruits that were artificially inoculated with fungi under laboratory-controlled condition.

The banana fruit were purchased from a local orchard in Guangzhou, China. After picking from the orchard, they were immediately transported to the lab. Banana fingers were selected with uniform maturity, shape, size, and visual absence of surface bruises and disease. The fingers were wiped with 75% of alcohol to sterilize the surface, then rinsed with sterile distilled water. All fruit were divided into control and infected groups.

*C. musae*, provided by Agricultural Culture Collection of China (Beijing, China), was used for the *in vitro* inoculation test. Infected samples were obtained by pricking the middle of the finger to a depth of approximately 1.5 cm using a steel needle with mycelium and spores. Samples in the control group were also pricked to 1.5 cm with a sterile inoculation steel needle. All samples were stored in an incubator at 28°C and 75% relative humidity (RH). Thirty samples in the infected group were selected for NIR spectra collection every 24 h. As the bananas had seriously rotted by the 11th day, the inoculated samples were only assessed on the first ten days in this study.

### 2.2 Spectroradiometer and data collection

The reflectance spectra collecting system was composed of a Vis/NIR spectrophotometer (USB2000, Ocean Optics, Dunedin, Florida), a stabilized halogen light source (HL2000, Ocean Optics, Dunedin, Florida), a Y-shaped fiber-optic reflectance probe, and a custom lifting platform. The visible–NIR spectrometer scanning range and sampling interval were 339–1019 nm and 0.29 nm, respectively. During acquiring spectral data, samples were placed on a custom lifting platform and manually adjusted to approach the probe. The integral time was set to 2.8 ms. One spectrum was obtained with 20 averaged scans, and the smoothing window point was set as 9 to reduce noise in the spectra. As shown above, the spectra were obtained for the control group and every 24 h for the infected groups. Consequently, a total of 30 spectra for the control group and 300 spectra for the infected group were achieved.

### 2.3 Primary chemometrics methods

In this work, traditional feature extraction methods (e.g., PCA) and distinct effective wavelength extraction algorithms were utilized to mine spectral feature information. SVM- and PLSDA-based models were established using the full-spectra, PCs, and effective spectral variables. Deep learning models (1D-CNN) were also constructed and compared.

#### 2.3.1 Feature selection methods

The PCA, PC loading coefficient, SPA, and CARS methods were adopted for spectral feature extraction. PCA can reduce the dimensions of the data, and get a glimpse of the patterns hidden in the full spectral data (Liu et al., 2010). Variable selection methods, such as PC loading coefficient, SPA, and CARS, can extract characteristic wavelengths in the original spectra, removing irrelevant and redundant information.

The PCA method that original spectral variables were projected into new orthogonal variables, known as principal components (PCs), was allowed for maximization of the sample variance (Yang et al., 2022). PCA can help to explore the spectral characteristics used for class separation (Munera et al., 2018). The wavelengths with larger absolute coefficients are considered necessary in the corresponding PC (Xing et al., 2010). Thus, the PC loading coefficients could help to determine the effective wavelengths.

SPA is a forward-loop variable selection algorithm. It starts with one wavelength and individually incorporates a new one at each iteration, until a specified number of wavelengths with the largest projection vector is reached (Araújo et al., 2001; Galvao et al., 2008). In other words, the number of wavelengths is determined by the minimum root mean square error (RMSE) values from the full internal cross-validation.

CARS combines Monte Carlo sampling with the partial least squares regression (PLSR) model. It selects N subsets of variables from N Monte Carlo (MC) sampling runs iteratively and competitively (Wang and Wang, 2022). The subset with the lowest RMSE of cross-validation (RMSECV) value is chosen as the optimal wavelength combi-nation. In every sampling phase, the spectral variables are selected by the adaptive reweighted sampling (ARS) method and the exponentially decreasing function (EDF), based on the regression coefficients of the PLS model (Zhang et al., 2019).

#### 2.3.2 Establishment of models

Traditional classification algorithms—including SVM and PLSDA methods—and deep learning algorithms—including the 1D-CNN—were employed in this study.

SVM is a supervised machine learning approach based on statistical learning theory. It projects data into a higher dimensional space and creates hyperplanes to separate each class (Pardo and Sberveglieri, 2005; Zhang et al., 2021). The higher-dimensional space is generally implemented using a kernel function, such as a Linear Function, Polynomial Function (Poly), Radial Basis Function (RBF), or Sigmoid Function (Sigmoid) (Kurosaki et al., 2020). SVM exhibits excellent performance in classifying high-dimensional data with limited training data.

PLS-DA is a supervised classification algorithm based on the principle of PLSR (Ivorra et al., 2013), which has also been widely applied in discriminant analysis. It projects the data matrix into a group of linear latent variables (LVs), then uses the PLS model combined with a suitable threshold to give the class number for each sample. During model training, the optimal number of LVs was determined according to the results of cross-validation (He et al., 2022).

A typical 1D-CNN consists of convolutional, pooling, and fully connected layers (Shen and Viscarra Rossel, 2021). It can also be understood as concatenating a feature extractor block with a classification head (Fazari et al., 2021). The convolutions, pooling operations, and non-linear activation functions belong to the feature extractor block. The convolutional layer can be used to realize feature mapping, which consists of a series of convolution kernels. The kernels are used to perform a convolution operation on a local data area, extracting the continuous features of different areas by sliding translation with a specific stride (Chai et al., 2021). In NIR spectral analysis, as the input vectors are one-dimensional spectral data, the feature map was calculated using one-dimensional convolution (Rong et al., 2020). The pooling layer can prevent overfitting by reducing the parameters of the CNN, and can also realize the spatial invariance of features while lowering the feature map. In addition, the activation function is used in a CNN to increase the non-linear capability of the network. The classification head is a fully connected classical neural network that produces a probability vector, which indicates the probability of the input data belonging to each class.

### 2.4 Model evaluation

The performance of all the models was evaluated in terms of sensitivity, precision, specificity, and accuracy. The sensitivity, specificity, and precision are used to evaluate a model’s classification performance for each group, while the accuracy is used to assess the overall performance of the model. Sensitivity and specificity are used to evaluate the capacity of a model to correctly recognize samples belonging to a specific group or reject samples from other groups (Jiang et al., 2022). Precision represents the ability of a model to correctly quantify the number of positive predictions (Tian et al., 2022). The accuracy denotes the ratio of the number of correctly classified samples to the total number of samples in a group. These parameters were calculated based on the confusion matrix, which comprises counts of true positive (TP), true negative (TN), false negative (FN), and false positive (FP) results. TP and TN represent that the samples are correctly classified as belonging or not belonging to a specific class, respectively. In contrast, FP and FN represent the samples incorrectly classified as belonging or not belonging to a given class, respectively (Kurosaki et al., 2020). The model evaluation parameters were calculated as follows:


(1) $$A\text{cccuracy}=\frac{TP+TN}{TP+FP+TN+FN}\times 100\%,$$

(2) $$Sensitivity=\frac{TP}{TP+FN}\times 100\%,$$

(3) $$Specificity=\frac{TN}{TN+FP}\times 100\%,$$

(4) $$Precision=\frac{TP}{TP+FP}\times 100\%,$$

## 3 Results and discussion

### 3.1 Preliminary level division and overview of the spectra

**Figure 1:**
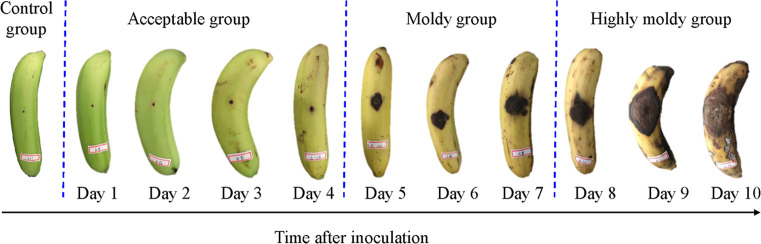
The appearance of infected banana fingers at different incubation time.

The RGB images of the control group and inoculated fruit of 1 to 10 days are shown in **Figure 1**. It can be seen that the symptoms of the fungal infection expanded gradually as the inoculation time increased. In the first four days, the banana was bright green, and the pricked regions were small. The samples in the control and acceptable group looked quite similar. Especially, there is no obvious difference in sample exterior between days 1 and 2 in the acceptable group and control group. From the fifth day, the symptom size sharply increased, and the color of the fruit turned yellow. The appearance of more yellow implies increased ripening. The symptoms further expanded from days 8 to 10, until they were severely rotted. To identify bananas infected with *C. musae* at different levels, all samples were tentatively divided into four classes—control, acceptable (days 1–4), moldy (days 5–7), and highly moldy (days 8–10)—according to the change of the symptom. A five-class dataset, including the control and day 1–4, was also prepared for identifying the fungi infection time at early stage.

**Figure 2:**
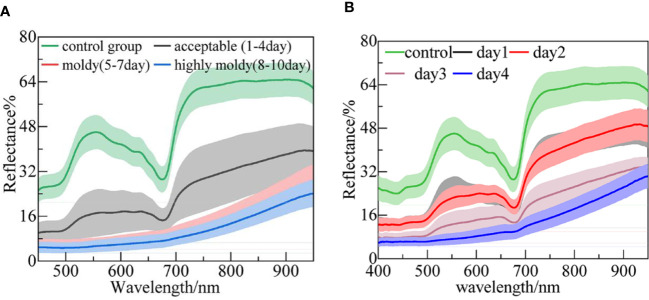
The mean spectra of banana fingers at different: **(A)** Infection levels; and **(B)** days post-infection.

The original average reflectance spectra of the four-class samples are shown in **Figure 2A**. The wavelength ranges of 339–400 nm and 950–1019 nm were removed to reduce noise caused by the lower sensitivity of the spectrometer. The shaded regions with different colors denote the deviation bands of the spectra. The mean spectrum of the control group (pricked with a sterile steel needle) presented a higher reflectance intensity. The overall trend of the acceptable group’s spectral profile was similar to that of the control group, while the reflectance intensities were relatively small. This is because the fungal infection caused a black disease spot and made the tissue more porous, causing more light to diffuse scattering, rather than reflecting (Liu et al., 2020). In addition, the peaks and valleys on the spectra represent some chemical composition or functional groups. The apparent peak at around 550 nm, as mentioned by Min et al. (2006), is related to nitrogen absorption. Furthermore, the regions in the 600–680 nm range represent anthocyanin and other pigments that are responsible for fruit coloration (ElMasry et al., 2007). Among them, the distinct valley around 675 nm can be attributed to chlorophyll. There was a sharp rise in the 680–740 nm band, which can be denoted as the ‘red edge’. The spectral pattern and reflectance values for the moldy and highly moldy groups obviously differed from those of the control and acceptable groups. In these two groups, the wave peaks and valleys became unclear as the reflection intensity further reduced. For example, in the moldy and highly moldy groups, the valley at 675 nm disappeared. With the increase in culture days, the changes in spectra agreed with those previously reported by Liu et al. (Liu et al., 2019). The difference in spectral profiles may be due to the changes of chemical composition and structural destruction of banana tissue caused by the fungal infection.

The average reflectance spectra of the five-class samples (i.e., control and days 1–4) are shown in **Figure 2B**. The patterns of the spectra are similar to those presented in **Figure 2A**. The highest reflectance appeared in the control group. It was difficult to distinguish the spectra between days 1 and 2. This may be because the fungi in the bananas were in a lag phase on these days, and the tissue structure had not been broken out (Sun et al., 2017). With the invasion of the fungi, the reflectance was further reduced, and the peak and valley were no longer obvious.

**Figure 3:**
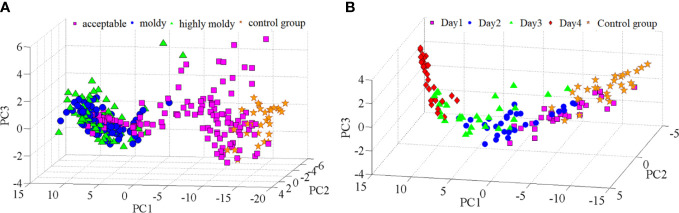
PC score plots: **(A)** Banana fingers with different fungi infection levels; and **(B)** banana fingers with different fungi infection time.

Principal component analysis (PCA) was carried out further to analyze the differences in spectra among the four-class sample. The scores of the first three PCs (**Figure 3A**) explained 92.13%, 5.25%, and 1.63% of the variance, respectively. It was found that samples in the four groups were distributed along the direction of PC1, and samples in the control and acceptable groups could be generally separated from those in the moldy and highly moldy groups. Similar results have also been observed for moldy peanuts and fungi-contaminated peaches, based on near-infrared spectroscopy (Liu et al., 2020; Shen et al., 2018). However, visual boundaries between the control and acceptable groups, as well as between the moldy and highly moldy groups, were missing. This motivated the use of chemometric techniques to improve the separation performance. Similar to the analysis of fungi infection levels, PCA was also performed for the five-class samples. The contribution rate of the first three PCs was 89.39%, 7.32% and 2.19%, which were accounted for >98% of the spectral variance. A three-dimensional (3D) score plot of those PCs was constructed and shown in **Figure 3B**. The samples in the control group and days 1–4 were also distributed along the direction of PC1, while the boundary of each class was not obvious.

### 3.2 Discriminant models based on traditional methods

#### 3.2.1 Spectral pre-treatment and division

In this study, the SVM and PLSDA methods were selected to establish models to discriminate bananas with different infection levels and time based on the four- and five-class datasets, respectively. As a pre-treatment process, the standard normal variate (SNV) was first applied to correct scatter caused by variations in the appearance of the disease spots (Li et al., 2020). For the SVM models, a polynomial kernel was selected as the kernel function, and the parameters of SVM were optimized by grid search method. A five-fold cross-validation were used during the construction of SVM models. Both the full spectral data and the PCs were taken as inputs of the SVM, and all the SVM inputs were normalized using the min–max method to improve the model performance. For the PLSDA models based on the full spectral data, five-fold cross-validation was also used. During modeling, the four- and five-class data sets were randomly divided into calibration and validation sets with a ratio of 3:1. The model performance was evaluated according to the accuracy in the calibration, cross-validation, and validation sets.

#### 3.2.2 Model construction based on full spectra

The performances of the Full-SVM, PCA-SVM, and Full-PLSDA models on the four- and five-class data sets are detailed in **Table 1**. During the construction of the PCA-SVM models, the proper input numbers of PCs was determined based on their accumulative contribution rates and the criterion of correct classification of the models (Ropodi et al., 2016). For the four-class model, a high model accuracy was achieved when the first 14 PCs were used. After that, the accuracies fluctuated little with an increasing number of PCs. Thus, the first 14 PCs were selected as input. The first 12 PCs were selected for the five-class PCA-SVM model, based on the same principles. For the PLSDA models, optimum input components (LVs) were selected based on the compromise between less input and lower error rate during model training and cross-validation. A total of 14 and 10 LVs were chosen for the four- and five-class PLSDA models, respectively. **Table 1** lists the accuracies of the PLS-DA and SVM models with their optimal parameters for comparison.

**Table 1:** Classification results of SVM and PLSDA models built with full spectra.

Model type	Modeling Method	Input variables	Classification accuracy (%)		
			Calibration set	Cross-validation set	Validation Set
Four-class model	Full-SVM	1645	96.76	96.76	91.57
	PCA-SVM	14 PCs	99.19	96.36	93.98
	PLSDA	14 LVs	97.98	96.76	93.98
Five-class model	Full-SVM	1645	97.32	91.07	92.10
	PCA-SVM	12 PCs	100	95.53	94.74
	PLSDA	10 LVs	95.54	92.86	92.11

It can be seen that all four- and five-class discriminant models achieved favorable results, with calibration accuracies over 95.54% and validation accuracies over 91.57%. The results indicated that the use of NIR spectra with proper modeling methods feasibly allows for identifying bananas with different fungi infection levels and time. For the Full-SVM models, the accuracies were 91.57% and 92.10% for the four- and five-class, respectively. However, large number of variables (full wavelengths of 1645 variables) resulted in complex computations during model construction. In the PCA-SVM and PLSDA models, the number of variables was remarkably reduced to a dozen. The PCA and PLSDA methods decompose the data into new, uncorrelated variables, reducing the number of variables in the model. New variables imply new directions in the pattern space, and they can explain as much variance as possible with respect to the raw full spectral data (Ciosek et al., 2005). This may be also the reason why the PCA-SVM and PLSDA models performed better than the full-SVM models, with validation accuracies of 93.98% and 93.98% for four-class models and 94.74% and 92.11% for five-class models, respectively. As the PCA-SVM models obtained the highest accuracies, they were selected for further analysis.

#### 3.2.3 Simplified models based on selected wavelengths

**Figure 4:**
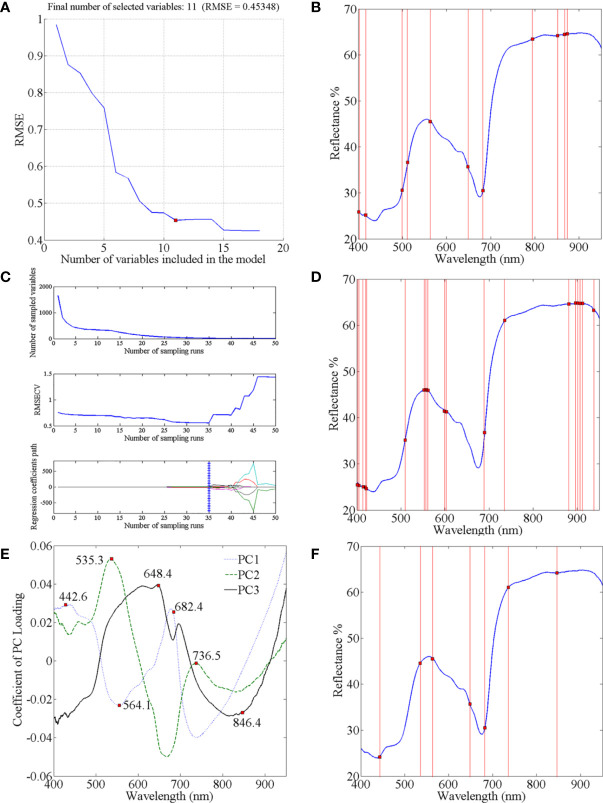
Selection of characteristic wavelengths using SPA CARS and PC Loading coefficient: **(A)** RMSE iteration decline curves of SPA; **(C)** process of CARS wavelength selection; **(E)** characteristic wavelengths selected by PC Loading coefficient; and location of characteristic wavelengths extracted by **(B)** SPA; **(D)** CARS; and **(F)** PC Loading coefficient.

Through spectral data analysis, selecting effective wavelengths can eliminate redundant information, retain critical information in the original data, and reduce the calculation burden. We considered three effective wavelength selection methods: SPA, CARS, and PC loading coefficient. Taking four-class data as an example to introduce the application of these methods. In the process of SPA, the RMSE iteration decline curves are shown in **Figure 4A**. The RMSE decreased dramatically with increasing number of wavelength variables, then fluctuated slightly when the value reached 11. Therefore, the number of effective wavelengths for the four-class models was determined to be 11 (**Figure 4B**). In the process of selecting variables by CARS (**Figure 4C**), the results are shown in **Figure 4D**. The number of sampled variables decreased rapidly at first with the increase of sampling time, then stabilized. Regarding the RMSECV values in the second sub-plots of **Figure 4C**, they also gently declined and remained stable when the sampling time was 35. This indicated that the uninformative variables were gradually eliminated, and the subsequent increase in the RMSECV was due to the removal of effective variables. Consequently, the optimal effective wavelength subset of variables was determined in the 35th sampling run. The loading lines for the PC1, PC2, and PC3 are shown in **Figure 4E**. The peaks and valleys correspond to greater absolute coefficient values, and the corresponding wavelengths were considered crucial (Xing et al., 2010). Thus, the seven wavelengths (442.6, 535.3, 564.1, 648.4, 682.4, 736.5, and 846.4 nm) from the PC loading were identified as the informative wavelengths for classifying the infection level (**Figure 4F**). Similarly, the effective wavelengths in the five-class spectra were also extracted using these three methods.

All the selected wavelengths are summarized in **Table 2**. For the two data sets, the effective wavelengths selected by the three methods were spread over the entire Vis–NIR wavelength range. The wavelengths chosen by CARS and SPA were similar and mainly concentrated in the regions of 400–700 nm and 850–900 nm. CARS select-ed a greater number of wavelengths, but some successive wavelengths were chosen, and bands were clustered around certain wavelengths (e.g., 402 nm, 555 nm, and 897 nm). Compared with CARS and SPA, the PC Loading coefficient method produced sparser distributions of effective variables in the full spectra. The above-mentioned selected wavelengths were subsequently used, instead of the original full spectra, to build new simplified SVM and PLS-DA models. The sample division method, kernel function and parameters optimized method for the simplified models were the same as those used in the modeling based on the full-spectra.

**Table 2:** Effective wavelengths selected by different methods.

Data set	Wavelength selection method	Number of wavelengths	Effective wavelength
Four-class	SPA	11	401.1, 416.7, 500.7, 512.7, 564.1, 648.4, 682.4, 795.9, 851.9, 867.4, 872.6
	CARS	18	402.2, 402.9, 416.7, 417.1, 417.8, 509.4, 554.2, 555.2, 555.9, 603.5, 603.8, 690.2, 881.5, 896.9, 897.3, 897.9, 898.5, 937.8
	PC Loading	7	442.6, 535.3, 564.1, 648.4, 682.4, 736.5, 846.4
Five-class	SPA	14	405.5, 412.2, 465.1, 507.6, 524.2, 617.7, 648.7, 745.7, 839.2, 859.1, 864, 869.9, 874.5, 945.7
	CARS	16	464.7, 465.4, 465.8, 500.4, 500.7, 501.5, 625, 625.7, 648.8, 649.1, 892.1, 892.7, 893.6, 899.1, 899.4, 899.7
	PC Loading	7	442.6, 535.3, 564.1, 652.6, 682.4, 736.5, 874.5

The prediction results are shown in **Table 3**. In both the four- and five-class data sets, the models based on SPA and CARS achieved acceptable classification accuracy of over 81%. These indicated that the wavelengths extracted by the SPA and CARS algorithms were efficient. The results of PC loading-based models were inferior to those of the SPA and CARS models. Of the wavelengths selected by PC Loading, some were also selected by CARS and SPA, indicating that this method could screen feature spectra information; however, as this method selected few variables, too much information may be lost, resulting in lower classification accuracy.

**Table 3:** Classification results of SVM and PLSDA models built with effective wavelengths.

Model type	Modeling Method	Wavelength selection method	Input variables	Classification accuracy (%)		
				Calibration set	Cross-validation set	Validation Set
Four-class model	SVM	SPA	11	97.16	93.12	91.57
		CARS	18	96.76	94.74	84.33
		PC Loading	7	82.19	82.19	77.11
	PLSDA	SPA	10 LVs	86.23	83.00	84.34
		CARS	9 LVs	92.31	91.90	81.93
		PC Loading	6 LVs	76.11	76.11	73.49
Five-class model	SVM	SPA	14	99.11	96.43	89.47
		CARS	16	100	88.39	81.58
		PC Loading	7	100	90.18	81.58
	PLSDA	SPA	9 LVs	96.43	95.54	86.84
		CARS	9 LVs	92.86	85.71	84.21
		PC Loading	6 LVs	82.14	76.79	81.58

On the other hand, combining with different wavelength selection methods, all SVM models slightly outperformed the PLSDA models, which were similar to those in the full-spectra models. Compared with all the models, SPA-SVM achieved the best performance, with accuracies of 91.57% and 89.47% in the validation sets of the four- and five-class datasets, respectively. Although they did not perform as satisfactorily as the full-spectra models, the number of wavelengths was significantly reduced (by 99.3% and 99.1%, respectively) in this step. In addition, the feature wavelengths in the spectral band of 600–680 nm may be related to certain colorants, which could be changed due to fungal infection. Wavelengths near 740 nm are assigned to the O–H stretching third overtone, while those near 842 and 899 nm are assigned to the C–H third overtone. Moreover, the wavelength 851.9 was near 850 nm, which is related to anthocyanin (Tian et al., 2019). The above aspects may explain why these wavelengths played an important role in the analysis.

### 3.3 Discriminant models established using 1D-CNN

**Figure 5:**
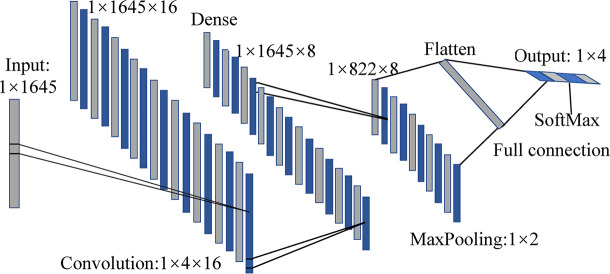
The structure of the 1D-CNN four-class model.

Based on the excellent performance in feature extraction and classification problems, 1D-CNN models based on full spectra were also established for comparison. For both four- and five-class discriminant pattern, calibration and validation sets were also divided by 3:1. Taking the four-class 1D-CNN model as an example, it consists of a convolutional layer, a max-pooling layer, a transition layer, and two fully connected layers, as shown in **Figure 5**. As the input spectral data were one-dimensional, the convolutional layer and pooling layer were also set to be one-dimensional. The convolutional layer used Rectified linear unit (ReLU) as an activation function, in order to increase the non-linear capability of the network. The kernel size, stride, and number of kernels were set as 4×1, 1, and 16, respectively. A fully connected layer was added after the convolutional layer to further extract features of the data. The number of output units was set as 8. A max-pooling layer was used for dimension reduction, configured with a pooling size of 2×1 and a stride of 2. The following transition layer (flatten layer) was used to tile the data in one dimension and realize the transition from the convolutional layer to the fully connected layer. The normalized exponential function (SoftMax) and cross-entropy were used in the last fully connected layer. The training set was divided into batches with a size of 32 to achieve rapid convergence of the model. The structure of the five-class 1D-CNN model was generally similar to that of the four-class model, except that the number of output units in the first fully connected layer was set as 16, and the batch size in the other fully connected layer was set as 64.

**Figure 6:**
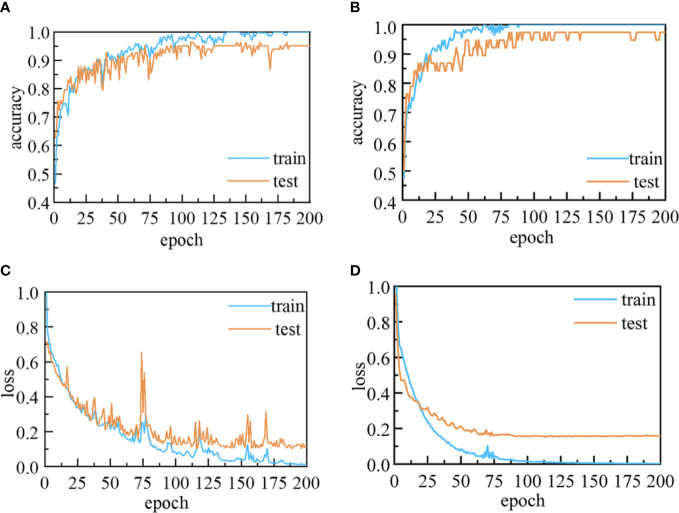
Accuracy and loss curves for the 1D-CNN models: **(A)** Accuracy and **(C)** loss curves for four-class 1D-CNN model; and **(B)** accuracy and **(D)** loss curves for five-class 1D-CNN model.

As shown in **Figure 6**, during the training and validation of the 1D-CNN models, the accuracies on the training and validation sets were increased, while the losses decreased with the increase in number of epochs. At the initial training, both the accuracy and the loss curve oscillate in a range respectively, which may be caused by the noisy in the spectra data and the inappropriate setting of learning rate. The use of backpropagation and gradient descent algorithms could help to calculate and adjust the weights of parameters and obtain the optimal solution of the model during the learning process. With the increase of training times and the optimization of parameters, the loss function gradually converges and the model becomes more stable. The validation accuracies in the last epoch increased to more than 95.18% and 97.73% for the four- and five-class data sets, respectively, while the corresponding losses declined to less than 0.12 and 0.16.

### 3.4 Comparisons of traditional models and 1D-CNN models

For comparison of the traditional and 1D-CNN models, **Tables 4**, **5** summarize the sensitivity, specificity, precision, and accuracy of the PCA-SVM, SPA-SVM, and 1D-CNN models for four- and five-class data sets, respectively.

**Table 4:** Classification results for the four-class data set obtained by traditional and 1D-CNN models.

Modeling Method	Input variables	Labels	Sensitivity (%)	Precision (%)	Specificity (%)	Accuracy (%)
PCA-SVM	14 PCs	Acceptable group	96.15	96.15	98.25	93.98
		Moldy group	85.71	90	96.77	
		Highly moldy group	96.97	94.11	96	
		Control group	100	100	100	
SPA-SVM	11 wavelengths	Acceptable group	93.10	96.43	98.15	91.57
		Moldy group	84.21	80	93.75	
		Highly moldy group	91.67	91.67	96.61	
		Control group	100	100	100	
1D-CNN	Full spectra (1645)	Acceptable group	100	100	100	95.18
		Moldy group	88	95.65	98.30	
		Highly moldy group	95.45	87.50	93.55	
		Control group	100	100	100	

**Table 5:** Classification results for the five-class data set obtained by traditional and 1D-CNN models.

Modeling Method	Input variables	Labels	Sensitivity (%)	Precision (%)	Specificity (%)	Accuracy (%)
PCA-SVM	12 PCs	Day 1	83.33	83.33	96.88	94.74
		Day 2	91.67	100	100	
		Day 3	100	100	100	
		Day 4	100	100	100	
		Control group	100	85.71	96.88	
SPA-SVM	14 wavelengths	Day 1	75.00	85.71	96.67	89.47
		Day 2	100	90.00	96.55	
		Day 3	100	100	100	
		Day 4	100	100	100	
		Control group	81.82	81.82	92.59	
1D-CNN	Full spectra (1645)	Day 1	90.90	100	100	97.37
		Day 2	100	87.50	96.77	
		Day 3	100	100	100	
		Day 4	100	100	100	
		Control group	100	100	100	

For the traditional models, the PCA-SVM models presented better performance than the SPA-SVM models, with an accuracy of 93.98% and 94.74% for the four- and five-class data sets, respectively. The PCs are comprehensive indices produced by linearly combining original spectral data (Su et al., 2021), eliminating correlations in the original data while preserving the variance in the raw data. While, SPA selects effective spectral variables directly from the 1645 variables. Eliminating a large number of variables may lead to the loss of some effective information. These may cause the prediction results of the SPA-SVM model to be slightly inferior.

From the results listed in **Tables 4**, **5**, it can be seen that the 1D-CNN models achieved the best results, with the highest accuracies of 95.18% and 97.37% for four- and five-class identification, respectively. The sensitivity, specificity, and precision were also found to be optimal for the 1D-CNN models. These encouraging results suggest that 1D-CNN models combined with NIR spectra have great potential in identifying bananas infected with the fungi at different levels and time. Compared with traditional methods, the models obtained satisfactory classification results without requiring the manual extraction of feature parameters, and could automatically extract more hidden features in the spectra. As mentioned by Tian et al. (2022), the convolutional layers in the 1D-CNN are equivalent to the operations of data pre-processing and feature extraction used in traditional machine learning approaches. As these layers were tuned by back-propagation, the optimization algorithm of deep learning could extract the hidden features in the spectra more accurately and effectively.

**Figure 7:**
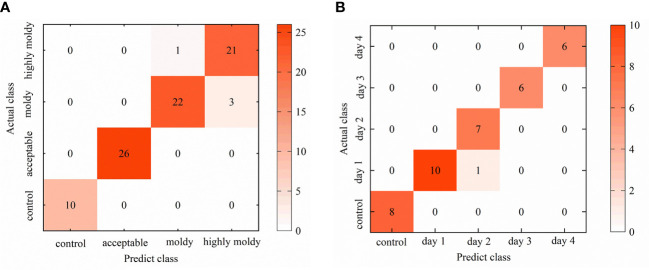
Visualized confusion matrix for: **(A)** Validation set of four-class 1D-CNN model; and **(B)** validation set of five-class 1D-CNN model.

Confusion matrixes for each 1D-CNN model were established, in order to further analyze the identification results (**Figure 7**). For the four-class model, the control and acceptable groups achieved better results, with 100% of individuals being well-classified. Thus, the stage of infection development can be precisely recognized, and separation between infected and uninfected fruit with great accuracy is possible. On the other hand, misidentification mainly occurred between the moldy and highly moldy groups. This may be caused by the slight differences in reflectance from the infected areas between these two groups, as black disease spots on the samples in these two groups were already obvious and the infected zone was rotten. These results are in agreement with the findings of Sun et al. (2018), who discriminated the degree of decay in peaches, based on the spectral range of 400–1000 nm.

The results for the five-class model confirmed that the days after inoculation could be separated with high accuracy using the 1D-CNN method. The 100% accuracy of the control group indicated that infected fruit could be accurately recognized after 24h. Some misclassification occurred when identifying days 1 and 2. To some extent, this is due to the fungi being in the lag phase withing the first few days, and the damage to banana tissue was not serious in this period. Consequently, the similarity of physical and chemical properties between samples in the first few days may lead to misclassification. These results were consistent with the findings of Siedliska et al. (2018), who identified the inoculation days of strawberries with BC fungi, and showed that the misclassification also more commonly occurred in the samples within the first few days. Sun et al. (2017) have discriminated peaches at different incubation time, and they also indicated that the very early diseased peaches (at 1 and 2 days) were in the same category. From the third day, the accuracy reached 100% as, over the first four days, symptoms gradually appeared with the ongoing infection, and the difference in the internal contents and exterior tissue surface gradually became obvious. The obvious spectral characteristics resulted in an accurate classification result.

## 4 Conclusions

This study tracked the growth and identified different infection stages of the *C. musae* in bananas using the Vis/NIR spectroscopy. Two types of discriminant models including 4-class and 5-class models were established using traditional methods, i.e combinations of three traditional feature extraction (SPA, CARS, and PC Loading) and two machine learning methods (PLSDA and SVM). A deep learning method of 1D-CNN was also used for comparison. The two models were used to examine the capability of NIR spectra in discriminating bananas infected at different levels (control, acceptable, moldy, and highly moldy), and different time at early stage (control and days 1-4), respectively. The models built by traditional methods had good performance with the detection accuracies in validation sets of 93.98% and 94.47% for 4- and 5-class models, respectively. The proposed 1D-CNN models can automatically extract the feature parameters and improved the detection accuracies, which were 95.18% and 97.37% for the 4- and 5-class discriminant models, respectively. These results demonstrated the feasibility of characterizing the process of C. *musae* infection in bananas using the Vis/NIR spectra. The resolution using Vis/NIR spectra in identifying bananas infected with *C. musae* can be accurate to 24 h. In addition, 11 and 14 effective wavelengths for the 4-class and 5-class models were selected using the traditional methods, which may serve as a simplified alternative for future practical implementation.

Additionally, it should be noted that this study preliminary analyzed the spectra change during the fungi infection process. To further characterize the infection mechanism of the fungi using the Vis/NIR, more physical and chemical changes, e.g. microstructure of tissue and chemical composition that are related to the changes of the spectral characteristics, will be analyzed combining with other methods such as physical and chemical examination, electron microscope scanning and fluorescence labeling in the near future.

## Data availability statement

The raw data supporting the conclusions of this article will be made available by the authors, without undue reservation.

## Author contributions

ZM, HF, and HL contributed to conception and design of the study. KZ, PM, and XC organized the database. XC, KZ, and HJ performed the statistical analysis. XC and HL wrote the first draft of the manuscript. ZM and HW provide the resources and supervised the experiment. All authors contributed to the article and approved the submitted version.
